# Efficacy and Safety of PD-L1 Inhibitors plus Chemotherapy versus Chemotherapy Alone in First-Line Treatment of Extensive-Stage Small-Cell Lung Cancer: A Retrospective Real-World Study

**DOI:** 10.1155/2022/3645489

**Published:** 2022-09-26

**Authors:** Jingjing Qu, Farhin Shaheed Kalyani, Qian Shen, Guangdie Yang, Tianli Cheng, Li Liu, Jianya Zhou, Jianying Zhou

**Affiliations:** ^1^Department of Respiratory Disease, Thoracic Disease Center, The First Affiliated Hospital, Zhejiang University School of Medicine, Hangzhou, Zhejiang 310003, China; ^2^The Clinical Research Center for Respiratory Diseases of Zhejiang Province, Hangzhou, Zhejiang 310003, China; ^3^Thoracic Medicine Department 1, Hunan Cancer Hospital, Affiliated Tumor Hospital of Xiangya Medical School of Central South University, Changsha, Hunan 410008, China; ^4^Lung Cancer and Gastroenterology Department, Hunan Cancer Hospital, Affiliated Tumor Hospital of Xiangya Medical School of Central South University, Changsha, Hunan 410008, China

## Abstract

**Background:**

Most patients with small-cell lung cancer (SCLC) have extensive-stage (ES) disease with a poor prognosis. Immunotherapy has shown good therapeutic effects in the treatment of ES-SCLC. We performed a real-world retrospective study to evaluate the safety and efficacy of PD-L1 inhibitors plus chemotherapy in patients with ES-SCLC.

**Method:**

A total of 224 patients diagnosed with ES-SCLC between March 2017 and April 2021 were included, of which 115 received only etoposide-platinum (EP) chemotherapy,and 109 received programmed cell-death ligand 1 (PD-L1) inhibitors and EP.

**Results:**

Immune checkpoint inhibitors (ICIs) plus platinum were associated with a significant improvement in overall survival (OS), with a hazard ratio (HR) of 0.60 (95% CI, 0.42–0.85; *P*=0.0054); median OS was 19 months in the ICIs plus EP group vs. 12 months in the EP group. The median progression-free survival (PFS) was 8.5 and 5.0 months, respectively (HR for disease progression or death, 0.42; 95% CI, 0.31–0.57; *P* < 0.0001). Male patients <65 years old, Stage IV, PS 0-1, without liver and brain metastasis had a better OS in the ICIs plus EP group than the EP group. The PFS and OS in the durvalumab plus chemotherapy group were insignificantly longer than that of the atezolizumab plus chemotherapy group. Any adverse effects (AEs) of grade 3 or 4 occurred in 50 patients (45.9%) in the ICIs plus EP group and 48 patients (41.7%) in the EP alone group. The most common immune-related AEs (irAEs) were immune hypothyroidism events (17.1%, 7/41), immune dermatitis (9.8%, 4/41), and immune pneumonia (9.8%, 4/41) in the durvalumab plus platinum-etoposide group. Immune liver insufficiency (10.3%, 7/68) and immune hypothyroidism (8.8%, 6/68) were the most common irAEs in the atezolizumab plus platinum-etoposide group.

**Conclusion:**

This study shows that adding PD-L1 inhibitors to chemotherapy can significantly improve PFS and OS in patients with ES-SCLC and demonstrates its safety without additional AEs.

## 1. Introduction

Lung cancer is a malignant tumor with the fastest increase in morbidity and mortality, especially in the past few years, threatening the health and quality of life worldwide [1–3]. Small-cell lung cancer (SCLC), also called small-cell carcinoma, accounts for almost 15% of all lung cancers. It is characterized by abnormal respiratory symptoms, early metastases, and poor prognoses [4, 5]. The dominant metastatic sites of SCLC are the contralateral lung, brain, pleural effusion, liver, adrenal gland, lymph nodes, and bone [6, 7]. The 5-year survival rate of SCLC remains at <7% [8, 9]. There is also a correlation with a high rate of gene mutation, including tumor protein p53 (TP53), retinoblastoma 1 (RB1), phosphatidylinositol-4,5-bisphosphate 3-kinase, catalytic subunit alpha (PIK3CA), and some rare oncogenic drivers [10–12]. Extensive-stage SCLC (ES-SCLC) presents in approximately two-thirds of all SCLCs and is characterized by its rapid growth rate, tumor extension that includes more than half the chest, metastasis to other parts of the body, or cannot be surrounded by standard radiotherapy [13–15]. Chemotherapy with etoposide and platinum (EP) has remained the standard first-line treatment in ES-SCLC [16, 17]. Median survival with this treatment is only 10 months, and most patients relapse within 4 months of accomplishing initial therapy [18, 19].

Cytotoxic T-lymphocyteantigen-4 (CTLA-4) was the first developed immune checkpoint inhibitor (ICI) [20]. Currently, the most common targets of ICIs are CTLA-4, programmed cell death 1 (PD-1), and programmed cell death ligand 1 (PD-L1) [21–24]. The development of immunotherapeutic approaches blocking the PD-1/PD-L1 pathway is a revolutionary breakthrough in treating lung cancer, including ES-SCLC [25–28]. A phase III clinical study using PD-1/PD-L1 inhibitors for treating ES-SCLC was first reported in 2016 [29]. Further phase III clinical trials have since been completed [30–34]. Anti-PD-1 agents (nivolumab or pembrolizumab) showed promising therapeutic effects for patients with ES-SCLC as a third-line or later-line treatment. However, the objective response rate (ORR) of PD-1 monotherapy is just 10–20% [35, 36]. A recent KEYNOTE-604 study reported that pembrolizumab combined with plus EP did not significantly improve OS compared with the placebo plus EP in patients with ES-SCLC [33]. Zhou et al. conducted a meta-analysis using ICIs plus EP to evaluate the therapeutic effect on ES-SCLC comprehensively. Compared with chemotherapy alone, ICIs plus chemotherapy significantly improved overall survival (OS) (hazard ratios (HR), 0.82; 95% CI, 0.75–0.89)) and progression-free survival (PFS) (HR, 0.81; 95% CI, 0.75–0.87) [37]. IMpower133 study group [30, 38] and CASPIAN investigators [32, 34] researched the role of PD-L1 inhibitors (atezolizumab or durvalumab) combined with chemotherapy on ES-SCLC. Both studies showed an increased OS in patients with ES-SCLC treated with PD-L1 inhibitors plus EP in contrast to EP alone. Thus, PD-L1 inhibitors could play a vital role in treating ES-SCLC. A retrospective multicenter study of the Turkish Oncology Group was shown by the median PFS and OS was 6.8 months (95% CI 5.7–7.8), and 11.9 months (95% CI 11–12.7) in extensive-stage SCLC patients who received atezolizumab combined with chemotherapy in a first-line treatment [39].

To the best of our knowledge, no useful data on PD-L1 inhibitors plus chemotherapy vs. chemotherapy alone in first-line therapy of ES-SCLC have been reported in the real world. Thus, we conducted this retrospective study to verify the efficacy and safety of anti-PD-L1 agents plus EP-based chemotherapy in ES-SCLC.

## 2. Method

### 2.1. Study Subjects and Treatment Procedure

Clinical data were collected from March 2017 to April 2021. The clinical study was conducted on 224 patients diagnosed as ES-SCLC by histology or cytology in the First Affiliated Hospital, Zhejiang University School of Medicine (*n* = 82) and Hunan Cancer Hospital, Affiliated Tumor Hospital of Xiangya Medical School of Central South University(*n* = 142). A comprehensive retrospective analysis was conducted. Patient demographics were collected, including clinical, pathologic, treatment, toxicity, and outcome data. According to the American Joint Committee on Cancer (7th *ed*), ES-SCLC is defined as stage IV or stage T3-4, owing to multiple lung nodules being too large or the tumor or lymph nodes being too large to be included in a tolerable radiation plan. According to the response evaluation criteria in solid tumors (RECIST) version 1.1 [40], patients are given an Eastern Cooperative Oncology Group (ECOG) performance status score to measure the severity of the disease and comprehensively evaluate whether the patient is treated with PD-L1 inhibitors. In addition, patient-related information was retrieved from the hospital medical records. This study was approved by the Institutional Review Board of the First Affiliated Hospital, Zhejiang University School of Medicine and Hunan Cancer Hospital, Affiliated Tumor Hospital of Xiangya Medical School of Central South University.

All patients received etoposide 100 mg/m^2^ (administered on days 1–3 of each 3-week cycle), and the carboplatin area under the curve was 5 mg/mL/min or cisplatin 75 mg/m^2^ (dosed on day 1 of each cycle). Among the included 224 patients, 115 received only etoposide and platinum-based chemotherapy, and 109 received PD-L1 inhibitors, including 1,000 mg of durvalumab or 1,200 mg of atezolizumab injected intravenously on day 1 of each 3-week cycle. During the period of clinical administration, patients continued to be cared for in accordance with conventional methods. The platinum-based chemotherapy was treated with four to six cycles. ICIs were used until one or more of the following conditions occurred: unacceptable toxicity, disease progression, or death (including the abnormal function of vital organs, severe drug allergic reactions), treatment refusal, or withdrawal from treatment due to other reasons (such as pregnancy, consciously poor efficacy, poor compliance). According to RECIST version 1.1, the researchers used enhanced computed tomography (CT) every two cycles to evaluate the patient's treatment effect and give corresponding treatment guidance.

### 2.2. Outcomes and Assessments

The primary endpoint of the study was OS (defined as the time between the date of administration and the date of death, or the last follow-up). The secondary endpoint was PFS (defined as the date from the start of treatment time to the date of discontinuation of treatment due to radiologically confirmed disease progression, intolerable side effects, or death), objective remission rate (ORR) (defined as the proportion of patients achieving complete remission [CR] or partial remission [PR]), and safety assessment. The study followed the common terminology criteria for adverse events (version 4.03) to assess drug treatment-related toxicity and conduct a comprehensive analysis. Serious adverse events (SAEs) were evaluated as any life-threatening AE resulting in death, hospitalization, or prolonging an existing hospitalization. The treating physicians identified immune-related AE (irAE) as requiring steroids to resolve.

### 2.3. Statistical Analysis

The Fisher's exact test was used for continuous variable comparison between groups (PD-L1 inhibitor plus EP chemotherapy group and EP chemotherapy group alone). The Kaplan–Meier curve and log-rank statistics were used to analyze the survival of each group. All statistical analysis was using GraphPad Prism 8 (GraphPad Software, San Diego, California, USA) software for graphing analysis. A statistically significant difference was referenced as a *P*-value < 0.05.

## 3. Results

### 3.1. Patient Screening Process

From March 2017 to April 2021, a retrospective clinical analysis was performed on 224 eligible patients diagnosed with ES-SCLC by histology or cytology. Among the 224 enrolled patients, there were 109 in the PD-L1 inhibitor plus EP-based chemotherapy cohort and 115 in the EP-based chemotherapy cohort. The demographic and clinical characteristics of patients with ES-SCLC are shown in Table 1. The median age of the PD-L1 inhibitor plus chemotherapy group and the chemotherapy group alone was 63 years (43–72 years) and 64 years (47–76 years), respectively. There was no significant difference in age between the two groups (*P*=0.528). Most patients were male (89.9%, 98/109) and had a smoking history (80.7%, 88/109). Nearly all initial diagnoses were identified as Stage IV (91.7%, 100/109) in the PD-L1 inhibitor plus platinum chemotherapy group. There were 27.5% (30/109), 24.8% (27/109), and 28.4% (31/109) of patients with central nervous system (CNS), liver, and baseline bone metastases, respectively, in the PD-L1 inhibitor plus platinum chemotherapy group. In addition, the PD-L1 inhibitor plus chemotherapy group accounted for 38.5% (42/109) of patients who received chest radiotherapy.

In the chemotherapy group, most patients were male (82.6%, 95/115), smokers (78.3%, 90/115), and were already in stage IV disease at the first diagnosis (92.2%, 106/115). Additionally, 23.5% (27/115), 28.7% (33/115), and 29.6% (34/115) had CNS, liver, and bone metastases, respectively. Moreover, 20.9% (24/115) in the chemotherapy-only group received chest radiotherapy. There was no significant correlation between the two groups for sex, age, ECOG performance status, smoking status, and CNS metastasis.

Among 224 patients receiving platinum-based chemotherapy, 93.3% (209/224) received carboplatin therapy, and 6.7% (15/224) received cisplatin therapy. A total of 96.3% (105/109) received more than four cycles of etoposide treatment in the ICIs plus platinum-based chemotherapy group. There were 37.6% (41/109) who took durvalumab and 62.4% (68/109) who took atezolizumab. In the platinum-based chemotherapy group, 93.9% (108/115) patients received at least four cycles of etoposide treatment, and 39.1% (45/115) patients received up to six cycles of platinum with etoposide treatment.

### 3.2. Evaluation of the Efficacy of ORR, PFS, and OS

**Figure 1:**
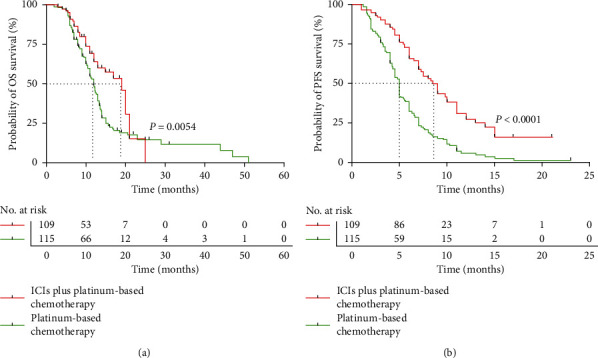
Overall survival (OS) and progression-free survival (PFS) in the ICIs combination chemotherapy cohort and only chemotherapy cohort. (a) Kaplan–Meier graph of OS in the two groups. (b) Kaplan–Meier graph of PFS in the two groups.

**Figure 2:**
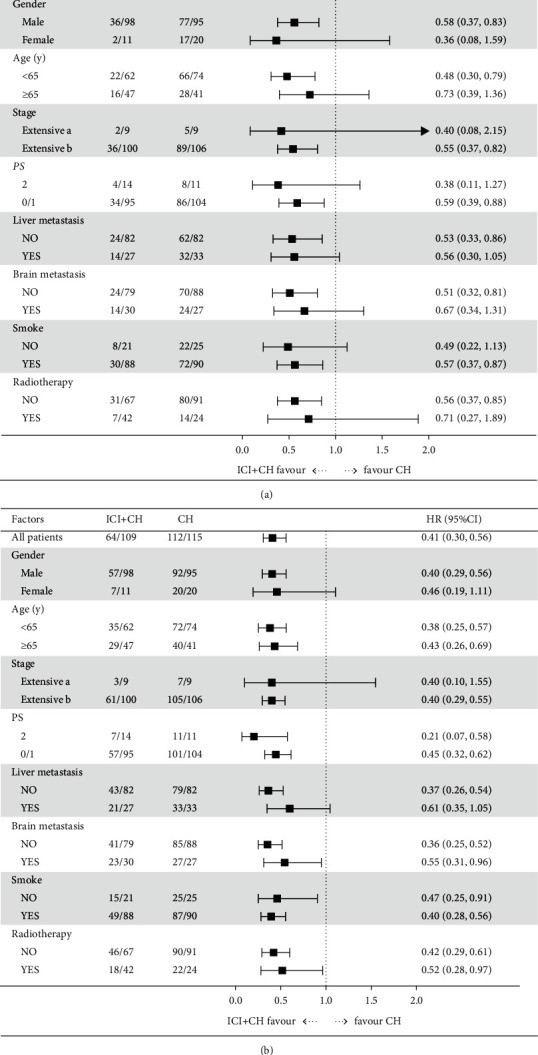
Forest plot of subgroup analysis of OS and PFS. (a) Forest plot of subgroup analysis of OS. (b) Forest plot of subgroup analysis of PFS. ICI: immune-checkpoint inhibitor; CH: chemotherapy; extensive a stage IIIb–IIIc; extensive b stage IV.

In this retrospective clinical study, the median follow-up time of patients' OS was 15.6 months (range 2.0–51.0 months). At data cut-off (August 30, 2021), the OS of the PD-L1 inhibitor plus platinum chemotherapy group (median 19.0 months) was significantly longer than that of the platinum chemotherapy group (median 12.0 months). The stratified hazard ratio (HR) for death was 0.60 (95% CI, 0.42–0.85; *P*=0.0054) (Figure 1(a)). In the PFS analysis, 58.7% (64/109) in the PD-L1 inhibitor plus platinum chemotherapy group and 97.4% (112/115) in the platinum chemotherapy group had disease progression. The PD-L1 inhibitor plus platinum-based chemotherapy group had longer PFS than the platinum-based chemotherapy group (8.5 vs. 5.0 months, respectively). The stratified HR for disease progression or death was 0.42 (95% CI, 0.31–0.57; *P* < 0.0001) (Figure 1(b)). In the PD-L1 inhibitor plus platinum-based chemotherapy group, the OS of male patients <65 years old, stage IV, PS0-1, smokers, without liver and brain metastasis was better than that of those in the platinum-based chemotherapy group (Figure 2(a)). Patients who have not received thoracic radiotherapy will benefit from PD-L1 inhibitor plus platinum-based chemotherapy. A subgroup analysis of PFS is given in Figure 2(b).

**Figure 3:**
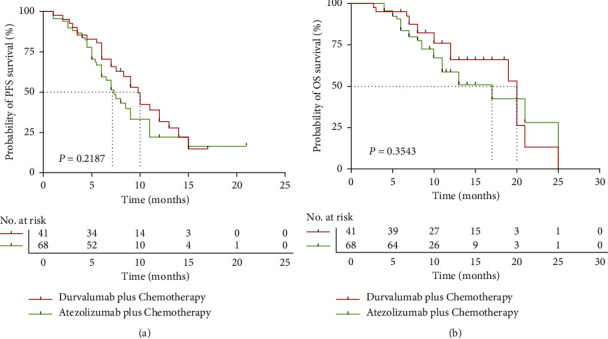
Overall survival (OS) and progression-free survival (PFS) in the durvalumab plus chemotherapy group and atezolizumab plus chemotherapy group. (a) Kaplan–Meier graph of PFS in the two groups. (b) Kaplan–Meier graph of OS in the two groups.

Furthermore, we compared the efficiency of PFS and OS between durvalumab and atezolizumab in the ICIs, combined with chemotherapy. There were 37.6% (41/109) patients who took durvalumab plus chemotherapy and 62.4% (68/109) who took atezolizumab plus chemotherapy. The durvalumab plus chemotherapy group had a longer PFS than the atezolizumab plus chemotherapy group (9.8 vs. 7.3 months, respectively) without significance. The stratified HR for disease progression or death was 0.75 (95% CI, 0.46–1.20; *P*=0.2187, Figure 3(a)). The OS of the durvalumab plus chemotherapy group (median 20.0 months) was also longer than that of the atezolizumab plus chemotherapy group (median 17.0 months). The stratified HR for death was 0.76 (95% CI, 0.41–1.40; *P*=0.3543). However, there was no statistical significance (Figure 3(b)).

Among the 109 patients evaluated in the PD-L1 inhibitor plus platinum chemotherapy group, 69.7% (76/109) achieved PR, 18.4% (20/109) achieved stable disease (SD), 6.4% (7/109) reached progressive disease (PD), and 5.5% of patients (6/109) could not be evaluated by the efficacy assessment. Among 115 patients evaluated in the platinum chemotherapy group, 68.6% (79/115) achieved PR, 14.8% (17/115) achieved SD, 13.1% (15/115) developed PD, and 3.5% (4/115) could not be evaluated using the efficacy assessment. The ORR of the PD-L1 inhibitor plus platinum chemotherapy group was 69.7%, while that of the platinum chemotherapy group was 68.6% (Table 2).

### 3.3. Evaluation for Safety

A total of 89.0% (97/109) of patients treated with PD-L1 inhibitor plus EP and 88.7% (102/115) of patients treated with EP had AEs of any cause or grade, as shown in Table 3. The 50 (45.9%) patients in the PD-L1 inhibitor plus EP group and 48 (41.7%) patients in the EP group had grade 3 or 4 AEs. In the PD-L1 inhibitor plus etoposide group, three patients (2.8%) died (one of neutropenia, one of pneumonia, and one of unknown cause), and five patients (4.3%) in the etoposide group died (two of pneumonia, one of septic shock, and two of respiratory failure).

Furthermore, we analyzed the difference in irAEs between durvalumab and atezolizumab. A total of 36.6% (15/41) in the durvalumab plus platinum-etoposide group and 29.4% (20/68) in the atezolizumab plus platinum-etoposide group experienced irAEs. The durvalumab plus platinum-etoposide group had 4.9% (2/41), and the atezolizumab plus platinum-etoposide group had 7.4% (5/68) grade 3 or 4 irAEs, respectively. The most common irAEs were immune hypothyroidism events (17.1%, 7/41), immune dermatitis (9.8%, 4/41), and immune pneumonia (9.8%, 4/41) in the durvalumab plus platinum-etoposide group. Immune liver insufficiency (10.3%, 7/68), immune hypothyroidism (8.8%, 6/68), immune dermatitis (5.9%, 4/68), and immune pneumonia (5.9%, 4/68) were the most common irAEs in the atezolizumab plus platinum-etoposide group (Table 4).

## 4. Discussion

To the best of our knowledge, this is the first comprehensive retrospective study analyzing differences between PD-L1 inhibitors combined with chemotherapy and chemotherapy alone on multiple survival indicators in patients with ES-SCLC. This research suggests that ICIs are suitable for treating ES-SCLC as first-line therapy. During the preliminary statistical analysis of PFS and the interim statistical analysis of OS, our study showed that compared with chemotherapy alone, adding ICIs to chemotherapy can significantly prolong OS (median of 19 months with adding PD-L1 inhibitors vs. a median of 12 months without adding PD-L1 inhibitors; HR, 0.60; 95% CI, 0.42–0.85) and PFS (8.5 months with adding PD-L1 inhibitors vs. 5.0 months without adding PD-L1 inhibitors; HR, 0.42; 95% CI, 0.31–0.57). In addition, improved OS was observed in subgroups of corresponding patients with ES-SCLC. Regarding the AEs occurring during the treatment period, the ICIs plus chemotherapy group had no significant increase in grade 3 or 4 AEs compared with the chemotherapy alone group. Zhou et al. reported that anti-PD-1/PD-L1 agents plus chemotherapy did not increase AEs when treating ES-SCLC [37]. Therefore, the combination of ICIs and chemotherapy does not cause more AEs. This also confirms that ICIs plus chemotherapy is relatively safe and may be conducive to prolonging the OS of patients with ES-SCLC.

In recent years, ICIs-based immunotherapy has been explored for treating ES-SCLC [41–43]. The clinical efficacy analysis result of the IMpower133 research group is a milestone in the treatment of ES-SCLC by ICIs-based immunotherapy [30]. Consistent with the main research results of atezolizumab combined with chemotherapy in the treatment of nonsquamous nonsmall cell lung cancer [44], atezolizumab combined with chemotherapy can improve the PFS (5.2 months in atezolizumab combined with chemotherapy vs. 4.3 months in chemotherapy; HR, 0.77; 95% CI, 0.62–0.96). Moreover, there were also significant improvements in OS (12.3 months vs. 10.3 months; HR, 0.70, 95% CI, 0.54–0.91) while treating ES-SCLC with atezolizumab [30]. The CASPIAN study showed the median OS to be consistent with previous reports [18, 19, 30]. CASPIAN investigators show that durvalumab plus chemotherapy significantly improved OS (13.0 months in durvalumab plus chemotherapy vs. 10.3 months in the chemotherapy alone group; HR, 0.73; 95% CI, 0.59–0.91) in treating ES-SCLC [32]. In our real-world research analysis, in patients receiving atezolizumab or durvalumab, the PFS and OS significantly increased. Our results, combined with other studies on atezolizumab or durvalumab plus carboplatin and etoposide [30, 32], further support ICIs plus chemotherapy as an effective first-line treatment for ES-SCLC.

However, our real-world research is a retrospective study, with clear differences between Inpower133 and the CASPIAN study. The CASPIAN study control group allowed up to six EP cycles, while the IMpower133 study control group allowed up to four EP cycles. In our study, 96.3% (105/109) in the ICIs plus platinum chemotherapy group took up to four cycles of platinum, and 39.1% (45/115) in the platinum chemotherapy group took up to six cycles. When designing the CASPIAN study, the guidelines recommended four to six EP cycles, but there is no evidence that six EP cycles produce better results than four. In addition, combining chemotherapy and immunotherapy lacks effective data concerning the safety in the treatment of ES-SCLC. Therefore, EP was restricted to the minimum recommended four cycles in the immunotherapy group. In contrast, in the control group, six cycles of etoposide were permitted to reflect current clinical practice. Using four or six cycles of EP in the ICIs plus chemotherapy group or chemotherapy alone group does not seem to affect the OS and PFS of patients with ES-SCLC. Due to the limited clinical studies, whether to use four or six cycles of EP requires further investigation.

ICIs (including anti-PD-1, anti-PD-L1, and anti-CTLA-4 agents) were largely studied for treating SCLC [25, 45, 46]. Zhou et al. conducted a meta-analysis to study the efficacy and safety of ICIs in treating patients with ES-SCLC [37]. They identified a total of six studies involving more than 2,900 patients. The result showed that compared with single chemotherapy, ICIs immunotherapy plus chemotherapy could significantly improve OS, and the difference in adverse events was not significant using PD-1 inhibitor or PD-L1 inhibitor [37]. These results indicate that ICIs plus chemotherapy can effectively alleviate patients with ES-SCLC. A recent KEYNOTE-604 study reported that PD-1 inhibitor pembrolizumab combined with plus EP did not significantly improve OS compared with the placebo plus EP in patients with ES-SCLC [33]. Therefore, the Food and Drug Administration (FDA) has approved these two combination drugs of PD-L1 inhibitors (atezolizumab and durvalumab) as the first-line treatment in ES-SCLC [47]. This further implies that ICIs may have a long-term therapeutic effect on treating patients with ES-SCLC. The specific immunotherapy mechanism needs to be further explored and evaluated with regard to the long-lasting effect.

In our study, the PFS and OS were longer in patients who received ICI therapy than in the Impower133 and CASPIAN studies. There may be multiple reasons for this: (1) Radiation therapy can prolong the OS and PFS for patients with ES-SCLC receiving ICI treatment. In the past two decades, thoracic oncologists have witnessed the widespread introduction of modern radiotherapy and chemotherapy in ES-SCLC, which has led to an improvement in the clinical status of radiotherapy and an increase in the number of related clinical studies [48–50]. Jeremic et al. [51] proved that chest radiotherapy is indispensable in treating patients with ES-SCLC after initial therapy. Ou et al. [52] retrospectively analyzed data from three counties of Southern California, USA. Among 3,428 patients with ES-SCLC, 1,204 (35.1%) received radiotherapy. This result showed that the median OS of patients who received radiotherapy was significantly higher than that of patients who did not (8.0 months vs. 4.0 months, *P* < 0.0001). An et al. [53] suggested that the three-year OS of the chemotherapy plus radiotherapy groups was significantly higher than that of the chemotherapy alone group (17.0 months in chemotherapy plus radiotherapy groups vs. 11.7 months in chemotherapy alone group, *P*=0.014) in a wide range of SCLC. In our study, the OS and PFS of the ICIs plus chemotherapy were longer than that of the prospective clinical trials. The reason is that 38.5% (42/109) in the ICIs plus chemotherapy cohort received radiotherapy in the real-world study. Radiation therapy was not performed in the Impower133 and CASPIAN study. (2) In addition, in our study, in patients with ICIs combined with chemotherapy, 27.5% (30/109) continued to choose ICIs in the second line or the back line after the progress of first-line immunotherapy, which may have prolonged the OS. (3) In retrospective studies, patients with poor efficacy may have been lost to follow-up.

Brain metastases are common and associated with poor clinical outcomes in ES-SCLC [54]. The role of prophylactic cranial irradiation (PCI) in patients with ES-SCLC after chemotherapy remains controversial, with conflicting evidence regarding its potential survival benefit [55, 56]. No significant difference in PFS or OS was observed in patients with brain metastases in the IMpower133 study group [30, 38] and CASPIAN investigations [32, 34]. In our study, only 7.3% and 5.2% received PCI in the ICIs plus chemotherapy and chemotherapy cohorts, respectively, when they showed clinical symptoms of brain metastases. The subgroup analysis shows that the OS in patients with brain metastases do have no obvious difference. Owing to the fewer patients with brain metastases, no confirmed conclusion can be drawn for PCI use in treating patients with ES-SCLC and brain metastases. Further investigation is required to define the effect of PCI in ES-SCLC, especially for patients using ICIs in combination with chemotherapy.

The overall AEs in the present study were similar between the two groups, including similar numbers of grade 3 or 4 AEs. The most common AEs were hematological toxicities and gastrointestinal symptoms. In addition, an increased number of AEs were observed in the chemotherapy group, possibly due to more patients receiving six cycles of EP in the chemotherapy alone group than in the ICIs plus EP group. Immune-related AEs were usually manageable with standard treatment guidelines [57]. In the present study, 32.1% (35/109) in the ICIs plus chemotherapy group experienced irAEs, and 6.4% (7/109) had grade 3 or 4 irAEs. This information implies that irAEs should not be ignored in future clinical studies.

**Figure 4:**
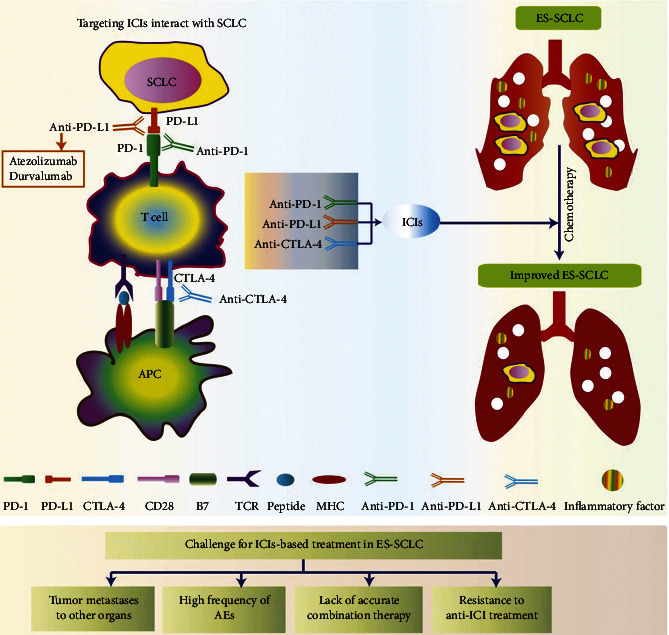
Targeting ICIs therapy for ES-SCLC and challenge for ICIs-based treatment in ES-SCLC. T cells blocked by anti-PD-1, anti-PD-L1, and anti-CTLA-4 agents, which interact with ES-SCLCs, and T cells medicate an effective role in killing ES-SCLCs. The current studies are limited by tumor metastases to other organs, high frequency of AEs, lack of accurate combination therapy, and resistance to anti-ICI treatment. ICI, immune checkpoint inhibitor; CTLA-4, cytotoxic T-lymphocyteantigen-4; PD-1, programmed cell death 1; PD-L1, programmed cell death ligand 1; SCLC, small-cell lung cancer; ES-SCLC; extensive-stage SCLC; APC, antigen presenting cell; TCR, T cell receptor; MHC, major histocompatibility complex; AEs, adverse events.

This clinical study has some limitations and challenges (Figure 4). First, a proportion of patients received radiation therapy, and its effect has not been analyzed in detail. Although some studies proved that thoracic radiotherapy plays an important role and can prolong the OS in patients with ES-SCLC [15, 58], some patients will choose not to undergo radiotherapy due to economic factors and poor compliance. Second, there is no safety and prognostic data regarding using PCI concurrently with ICIs. Further trials are needed to investigate the role of immunotherapy in patients with ES-SCLC who experience brain metastases. Third, SCLC is prone to distant metastasis, and we should explore more biomarkers to predict distant metastasis of SCLC. Forth, the mechanism and potential therapeutic measures of immunotherapy resistance in SCLC need to be explored.

## 5. Conclusion

This world study shows that adding PD-L1 inhibitors to chemotherapy can significantly improve PFS and OS in patients with ES-SCLC, and it shows the safety without additional AEs. Although more follow-up studies are needed to standardize clinical programs, this real-world study further demonstrates that platinum-based chemotherapy plus ICIs can play a vital role in treating patients with ES-SCLC.

## Figures and Tables

**Table 1:** Baseline of patients' demographics and clinical characteristics.

	ICIs plus platinum-basedchemotherapy (*n* = 109)	Platinum-basedchemotherapy (*n* = 115)	*P* value
Sex			0.125
Male	98 (89.9%)	95 (82.6%)	
Female	11 (10.1%)	20 (17.4%)	

Median age, years	63 (43–72)	64 (47–76)	0.528

Age group, years			0.275
＜65	62 (56.9%)	74 (64.3%)	
≥65	47 (43.1%)	41 (35.7%)	

Disease stage			＞0.999
IIIb–IIIc	9 (8.3%)	9 (7.8%)	
IV	100 (91.7%)	106 (92.2%)	

ECOG performance status			0.526
0-1	95 (87.2%)	104 (90.4%)	
2	14 (12.8%)	11 (9.6%)	

Smoking status			
Never smoker	21 (19.3%)	25 (21.7%)	0.741
Smoker	88 (80.7%)	90 (78.3%)	

CNS metastases			
No	79 (72.5%)	88 (76.5%)	0.541
Yes	30 (27.5%)	27 (23.5%)	

Liver metastases			
No	82 (75.2%)	82 (71.3%)	0.548
Yes	27 (24.8%)	33 (28.7%)	

Bone metastases			
No	78 (71.6%)	81 (70.4%)	0.884
Yes	31 (28.4%)	34 (29.6%)	

Thoracic radiotherapy			
No	67 (61.5%)	91 (79.1%)	0.005
Yes	42 (38.5%)	24 (20.9%)	

CNS: central nervous system; ECOG: eastern cooperative oncology group.

**Table 2:** The analysis of response rate and disease progression between ICIs plus platinum-based chemotherapy and platinum-based chemotherapy.

	ICIs plus platinum-basedchemotherapy (*n* = 109)	Platinum-basedchemotherapy (*n* = 115)
Confirmed objective response	76 (69.7%)	79 (68.6%)

Best objective response		
Complete response	0	0
Partial response	76 (69.7%)	79 (68.6%)
Stable disease	20 (18.4%)	17 (14.8%)
Progressive disease	7 (6.4%)	15 (13.1%)
Not evaluate	6 (5.5%)	4 (3.5%)

**Table 3:** Adverse events in the ICIs plus platinum-etoposide group and platinum-etoposide group.

	ICIs plus platinum-based chemotherapy (*n* = 109)		Platinum-based chemotherapy (*n* = 115)	
	Any grade	Grade 3 or 4	Any grade	Grade 3 or 4
Any event	97 (89.0%)	50 (45.9%)	102 (88.7%)	48 (41.7%)
Any event leading to death	3 (2.8%)		5 (4.3%)	

Adverse events in each group				
Neutropenia	56 (51.4%)	30 (27.5%)	67 (58.3%)	35 (30.4%)
Nausea	76 (69.7%)	32 (29.4%)	76 (66.1%)	27 (23.5%)
Vomiting	64 (58.7%)	37 (33.9%)	69 (60.0%)	44 (38.3%)
Anemia	58 (53.2%)	22 (20.2%)	60 (52.2%)	31 (27.0%)
Decreased appetite	37 (33.9%)	10 (9.2%)	40 (34.8%)	22 (19.1%)
Asthenia	23 (21.1%)	8 (7.3%)	17 (14.8%)	8 (7.0%)
Pneumonia	10 (9.2%)	2 (1.8%)	8 (7.0%)	1 (0.9%)
Decreased platelet count	17 (15.6%)	4 (3.7%)	10 (8.7%)	2 (1.7%)
Constipation	23 (21.1%)	8 (7.3%)	25 (21.7%)	9 (7.8%)
Diarrhea	19 (17.4%)	7 (6.4%)	16 (13.9%)	6 (5.2%)
Hypertension	8 (7.3%)	1 (0.9%)	10 (8.7%)	1 (0.9%)
High cholesterol	12 (11.0%)	4 (3.7%)	4 (3.5%)	0

**Table 4:** irAEs in the durvalumab plus platinum-etoposide group and atezolizumab plus platinum-etoposide group.

	Durvalumab plus platinum-based chemotherapy (*n* = 41)		Atezolizumab plus platinum-based chemotherapy (*n* = 68)	
	Any grade	Grade 3 or 4	Any grade	Grade 3 or 4
Any event	15 (36.6%)	2 (4.9%)	20 (29.4%)	5 (7.4%)

irAEs in each group				
Immune hypothyroidism	7 (17.1%)	0	6 (8.8%)	1 (1.5%)
Immune dermatitis	4 (9.8%)	0	4 (5.9%)	0
Immune pneumonia	4 (9.8%)	1 (2.4%)	4 (5.9%)	2 (2.9%)
Immune liver insufficiency	2 (4.9%)	1 (2.4%)	7 (10.3%)	1 (1.5%)
Immune nephritis	1 (2.4%)	0	0	0
Immune gastroenteritis	0	0	2 (2.9%)	1 (1.5%)
Immune hyperthyroidism	0	0	1 (1.5%)	0
Immune peripheral neuritis	0	0	1 (1.5%)	0

## Data Availability

The data are available from the corresponding author on reasonable request.

## Abbreviations

SCLC: Small-cell lung cancer
TP53: Tumor protein p53
RB1: Retinoblastoma 1
PIK3CA: Phosphatidylinositol-4,5-bisphosphate 3-kinase, catalytic subunit alpha
ES-SCLC: Extensive-stage SCLC
EP: Etoposide and platinum
CTLA-4: Cytotoxic T-lymphocyte antigen-4
ICI: Immune checkpoint inhibitor
PD-1: Programmed cell death 1
PD-L1: Programmed cell death ligand 1
ORR: Objective response rate
OS: Overall survival
HR: Hazard ratios
PFS: Progression free survival
RECIST: Criteria of evaluation criteria in solid tumors
ECOG: Eastern cooperative oncology group
CT: Computed tomography
CR: Complete remission
PR: Partial remission
CNS: Central nervous system
SD: Stable disease
PD: Progressive disease
AEs: Adverse events
irAEs: Immune-related adverse events
FDA: Food and drug administration.
